# Persistent lymphatic filariasis transmission seven years after validation of elimination as a public health problem: a cross-sectional study in Tonga

**DOI:** 10.1016/j.lanwpc.2025.101513

**Published:** 2025-03-20

**Authors:** Harriet L.S. Lawford, ‘Ofa Tukia, Joseph Takai, Sarah Sheridan, Selina Ward, Holly Jian, Beatris Mario Martin, Reynold ‘Ofanoa, Colleen L. Lau

**Affiliations:** a University of Queensland Centre for Clinical Research (UQCCR), Faculty of Health, Medicine, and Behavioural Sciences, The University of Queensland, Brisbane, QLD 4029, Australia; b Public Health Division, Ministry of Health, Nuku’alofa, Tonga; c National Centre for Immunisation Research and Surveillance of Vaccine Preventable Diseases, Sydney, NSW, Australia

**Keywords:** Kingdom of Tonga, Lymphatic filariasis, Post-validation surveillance, Elimination, Neglected tropical diseases

## Abstract

**Background:**

The World Health Organization (WHO) has validated 21 countries as having eliminated lymphatic filariasis (LF) as a public health problem. Post-validation surveillance (PVS) is required in countries where LF has been eliminated. Tonga eliminated LF in 2017, but no PVS strategy has been established. We aimed to identify any persistent LF transmission in Tonga in 2024 and provide evidence to support a PVS strategy.

**Methods:**

A four-pronged, targeted, cross-sectional study was conducted in the Tongan districts of Tongatapu, Ha’apai, and Ongo Niuas in May–July 2024 in communities, primary schools, high schools, and an outpatient clinic. Participants were tested for LF antigen (Ag) and microfilariae (Mf). The outcome measure for persistent LF transmission was Ag-positivity.

**Findings:**

Between 9 May and 19 July 2024, 1787 participants were recruited from 12 communities, 11 primary schools, five high schools, and one outpatient clinic. Overall, 39 participants (2·2%) were Ag-positive and five (0·3%) were Mf-positive. The highest Ag prevalence was in communities (4·0%; 95% confidence interval [95%CI]: 2·9–5·6), where all Mf-positives (n = 5) were identified. Ag-positivity was associated with male sex (adjusted odds ratio [aOR]:4·86; 95%CI: 2·25–10·46), older age (>50 years vs 5–10 years [aOR:7·51; 95%CI: 2·13–26·47]), and residing in Ha’apai (aOR:15·08; 95%CI: 5·41–42·05) and Ongo Niuas (aOR:10·85; 95%CI: 3·91–30·08).

**Interpretation:**

We found persistent LF transmission in Tonga seven years post-validation. Community surveillance yielded the highest Ag and Mf prevalence. Efficiency of PVS could be improved by integrating surveillance activities into the existing health system and conducting community-based surveys, particularly among older males and in high prevalence areas.

**Funding:**

Task Force for Global Health, 10.13039/100000865Bill & Melinda Gates Foundation, and the 10.13039/100000200United States Agency for International Development.

**Research in context**

**Evidence before this study**

We did a literature search in PubMed in September 2024 using the keywords “lymphatic filariasis”, “elimination”, and “post-validation surveillance”. Lymphatic filariasis (LF) was first targeted for elimination as a public health problem in 1997 at the World Health Assembly, with specific aims to eliminate LF by 2020 outlined by The Global Programme to Eliminate Lymphatic Filariasis during its inception in 2000. The *WHO Roadmap for Neglected Tropical Diseases 2021–2030* now aims for 81% of endemic countries to be validated as having eliminated LF as a public health problem, and for all countries where LF is eliminated to be implementing post-validation surveillance (PVS) by 2030. Limited guidance on ongoing surveillance after elimination of LF as a public health problem have been released to date. There is a need for effective, contextually-relevant PVS strategies to be conducted to identify any residual LF transmission so that resurgence is curtailed.

**Added value of this study**

This is the first published study on PVS of LF in Tonga and the first LF surveillance study conducted in Tonga since 2015. Our findings demonstrate persistent LF transmission in Tonga seven years since LF was eliminated as a public health problem. We found that community surveillance yielded the highest Ag and Mf prevalence, especially among older males.

**Implications of all the available evidence**

The Pacific Island Countries and Territories (PICTs) are leading the way globally in LF elimination efforts, with several countries approaching 10 years since validation of LF elimination as a public health problem. Countries where LF is eliminated need to have clear guidance about next steps once signals of persistent LF transmission have been identified. PICTs will be at the forefront in generating evidence around what PVS strategies work in the region and more broadly. We argue for a sustainable, contextually-appropriate PVS system that includes older males in community settings that can be integrated into the existing health system.

## Introduction

Lymphatic filariasis (LF) is a mosquito-borne disease caused by three species of filarial nematodes: *Wuchereria bancrofti* (responsible for 90% of cases), *Brugia malayi*, and *Brugia timori*. Following leprosy, LF is the second most prevalent cause of permanent deformity and disability worldwide.^1^ Though the majority of LF infections are subclinical, damage to the lymphatic system can occur causing lymphedema and, in severe cases, grade III lymphedema (also known as elephantiasis) and scrotal swelling (hydrocele).^2^

LF was first targeted for elimination as a public health problem in 1997 at the World Health Assembly; following this The Global Programme to Eliminate Lymphatic Filariasis was established in 2000, which resolved to eliminate LF by 2020.^3^ The *World Health Organization (WHO) Roadmap for Neglected Tropical Diseases 2021–2030* now aims for 58 of 72 (81%) countries to be validated as having eliminated LF as a public health problem, and for all 72 countries to have completed their mass drug administration (MDA) programmes and be implementing post-MDA or post-validation surveillance by 2030.^4^

**Fig. 1:**
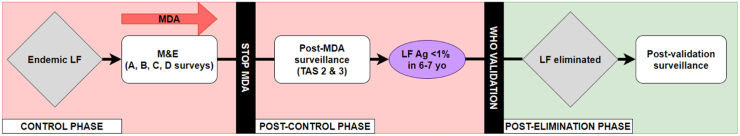
**Programmatic steps taken to achieve validation of lymphatic filariasis elimination as a public health problem by the World Health Organization.** Ag = antigen; LF = lymphatic filariasis; M&E = monitoring and evaluation; MDA = mass drug administration; WHO = World Health Organization; TAS = transmission assessment survey; yo = years old.

At a country-level, elimination of LF as a public health problem is validated by the WHO using the results of a series of Transmission Assessment Surveys (TAS).^5^ An antigenemia prevalence of less than 1% in 6–7-year-old children (in areas where *W. bancrofti* is endemic and *Aedes* is the primary vector^6^) is required in all evaluation units for a country to be validated (Fig. 1). Once achieved, countries are encouraged to conduct post-validation surveillance (PVS); the aim of PVS is to ensure that no resurgence of LF has occurred and prevalence has remained below target thresholds. Limited guidance has been released by WHO to guide countries on how to implement PVS, the surveillance strategies that should be adopted, and the thresholds that determine whether elimination as a public health problem has been sustained.

The Kingdom of Tonga, an island nation in the south Pacific, achieved validation of elimination of LF as a public health problem in 2017 after successfully recording antigen (Ag) prevalence <1% in all three TAS.^7^ The Tonga Operational Research for Post-validation Surveillance for Elimination of Lymphatic Filariasis (TOPELF 2024) study was designed in response to national concerns that LF resurgence may be occurring undetected in Tonga, particularly as no community surveillance for LF had been conducted since TAS-3 in 2015.

The aim of TOPELF 2024 was to identify any persistent LF transmission in Tonga and provide evidence to support a PVS strategy. The study was co-designed between the Tonga MOH and The University of Queensland and implemented using culturally appropriate methods. The objectives included:

1. To estimate the prevalence of microfilariae (Mf) and Ag in targeted communities and sub-populations,
2. To describe the demographic characteristics and geographical distribution of Ag-positive individuals (if found), and
3. To investigate different approaches for PVS of LF and provide an evidence base for developing ongoing PVS strategies in Tonga and regionally.

## Methods

### Ethics approval

The study was approved by the National Health Ethics and Research Committee of the Tonga Ministry of Health (Ref#20240129) on 22 February 2024 and ratified by The University of Queensland’s Human Research Ethics Committee (Project#2024/HE000493) on 12 March 2024. Processes for obtaining consent have been described.^7^ Briefly, written consent was sought from participants (parent/guardian consent for children aged <18 years), who were advised that they could revoke their consent at any time without any prejudice. Written information, consent forms, and surveys were provided/conducted by local field teams in a culturally sensitive manner in Tongan or English. The details and location of Ag-positive cases were passed to Tonga Ministry of Health (MOH) to ensure follow-up and treatment.

### Study setting

The Kingdom of Tonga is an archipelago of 176 islands stretching approximately 800 km from North to South. There are five administrative divisions: Tongatapu, ‘Eua, Ha’apai, Vava’u, and the isolated island group of Ongo Niuas.^8^ Thirty-six islands are inhabited by a population of approximately 100,179, of whom 70% reside on Tongatapu, the largest island that includes the capital city, Nuku’alofa.^9^ Tonga has a tropical climate with mean annual temperatures ranging from 23 to 28 °C.^8^

### Survey design

The study protocol, co-designed with the Tonga MOH, has been published.^7^ The protocol aimed to target communities and sub-populations at high-risk of LF infection by using local knowledge and historical survey data. Drawing on the understanding that older males and ‘never treated’ populations are at higher risk of LF infection,^10^^,^^11^ a four-pronged study was designed including surveys of 1) communities, 2) primary schools, 3) high schools (and boarding schools), and 4) an outpatient clinic. Individuals who approached the study team requesting to be tested were included as ‘volunteers’.

Six primary schools that recorded Ag-positive children in 2011 TAS-2 were considered ‘high-risk’, and the communities where these schools were located considered ‘high-risk’ communities. An additional ‘high-risk’ community and its primary school were selected due to high Ag prevalence recorded in the 2004 mid-term (B) survey. Four primary schools and communities that recorded no Ag-positive children in 2011 TAS-2 or the pre-stop MDA (C) survey in 2006 were selected as ‘low-risk’ sites and were considered reference populations against which LF Ag prevalence from ‘high-risk’ sites could be compared. Given the challenge and expense of travelling to Niuatoputapu in the remote Ongo Niuas island group, an additional community (Vaipoa) was included in the survey following requests by Tonga MOH. This ensured that majority of the population of Niuatoputapu island could be tested at the same time and thus negate the need for additional travel and testing later.

Boarding schools were selected because they provide a unique opportunity to test students from remote, outer island communities that are difficult and costly to access and may otherwise be missed. A high school in the Ongo Niuas island group was also included. The diabetes outpatient clinic at Vaiola Hospital in Nuku’alofa (the largest hospital in Tonga) was selected for the clinic-based component of the study due to large number of patients who could be recruited.

Any Ag-positive students or clinic patients were followed-up at home and all household members ≥5 years old were offered testing.

**Fig. 2:**
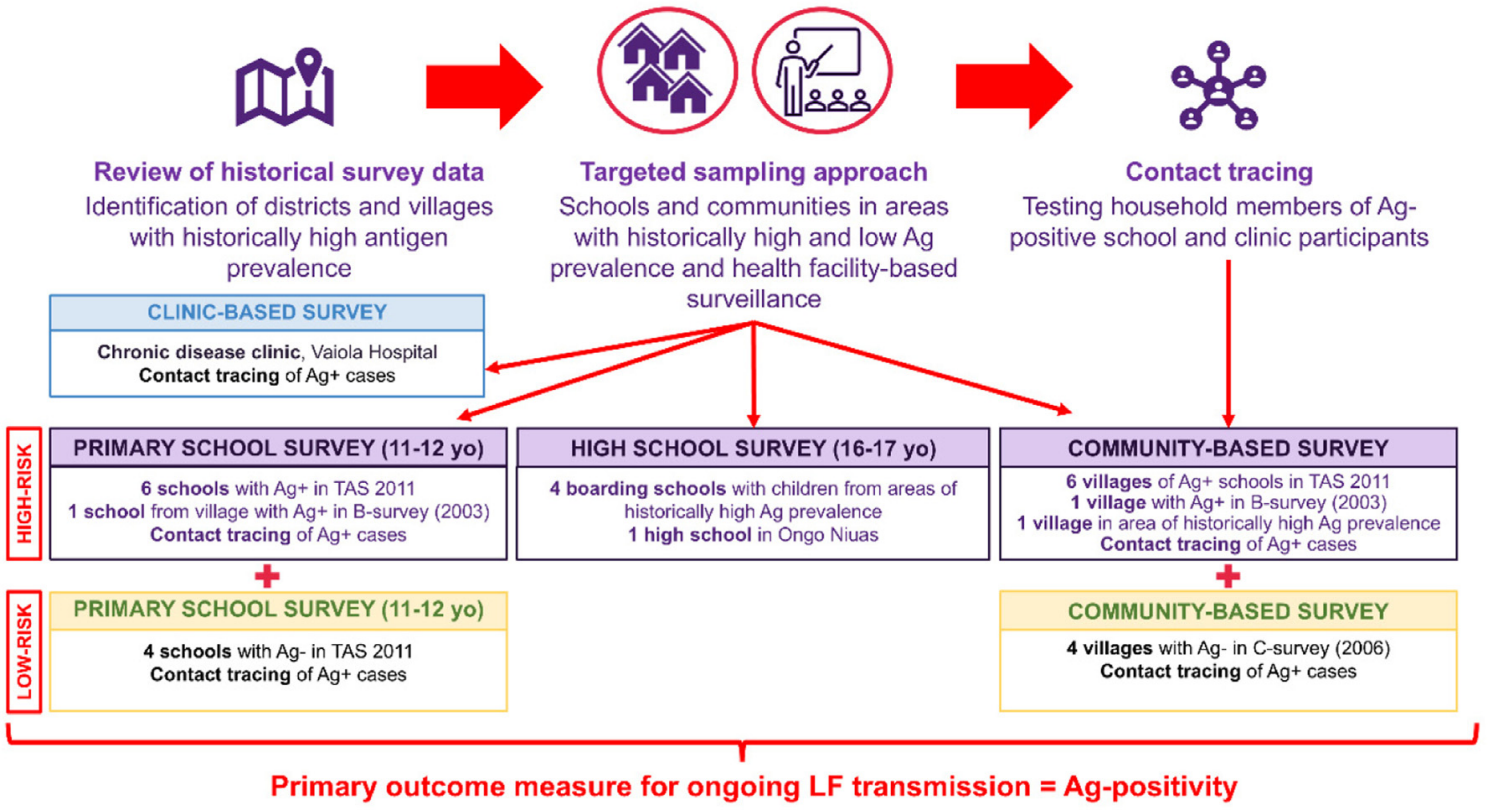
**Schematic illustrating the four-pronged study design of Tonga Operational Research for Post-validation Surveillance for Elimination of Lymphatic Filariasis 2024.** Following review of historical survey data, ‘high-risk’ (purple boxes) and ‘low-risk’ (yellow boxes) primary schools and communities were selected. One high school and five boarding schools were also considered ‘high-risk’. A clinic-based survey of diabetes patients was included (blue box). The primary outcome measure was antigen-positivity. Ag+: antigen-positive; Ag-: antigen negative; LF: lymphatic filariasis; TAS: Transmission Assessment Survey; yo: years old.

The study design is summarised in Fig. 2.

### Timing of survey

Fieldwork for TOPELF 2024 was conducted in two stages: 1) from 9 May to 6 June 2024 (Tongatapu and Ha’apai) and 2) from 11 to 19 July 2024 (Ha’apai and Ongo Niuas).

### Target sample size

The published protocol details participant selection and target sample size.^7^ Following consultation with the Tonga MOH, Vaipoa community (n = 75 participants) was included, bringing the total target sample size to 1955 (Supplementary Table S1).

### Household selection

As described,^7^ 15 households and five backup households in ‘high-risk’ and ‘low-risk’ communities were randomly selected by the Tonga Statistics Department using a random number generator. Backup households were selected in case selected households were uninhabited or inhabitants could not be reached. All household members aged ≥ 5 years were eligible and invited to participate. Individuals were considered a household member if the house was their primary place of residence (‘mostly sleep in this house’) or if they slept there the previous night.

### Data and sample collection

Electronic questionnaires were administered using EpiCollect5 software (https://five.epicollect.net/; V76.1.0) on smartphones. Questionnaires collected demographics (age, sex, country/place of birth, village of residence, occupation) and travel history in the past 12 months (international and within Tonga). GPS coordinates of households and schools were recorded using smartphones where possible. Enrolled participants had 300–400 μL of blood collected by finger prick into heparin-coated BD Microtainer® Blood Collection Tubes for Ag testing using Alere™ Filariasis Test Strips (FTS) (Abbott, Scarborough, ME), to make dried blood spots (DBS) using Cellabs Tropbio Filter Paper Blood Collection Disks™ (all participants), and to make slides for parasitological identification of Mf (Ag-positive participants only).

### Laboratory analysis

The primary outcome measure for persistent LF transmission was Ag-positivity, diagnosed using FTS. Mf slides were prepared from Ag-positive samples according to WHO guidelines.^12^ Slides were examined independently for Mf by two trained parasitologists in Australia. Participants were considered Mf-positive if Mf were observed in one or both slides. All participants had DBS made irrespective of Ag-positivity.

To enable linkage of demographic and laboratory data, participants were given a unique identifying number that was printed as QR code stickers and attached to consent forms, questionnaires, blood collection tubes, FTS, slides, and DBS.

### Statistical analysis

Data were analysed using Stata statistical software (StataCorp, Version 18.0, College Station, TX). Summary statistics with 95% logit-transformed confidence intervals (95% CI) for Ag and Mf prevalence were calculated by setting (community, primary school, high school, clinic, volunteers) and island group (Tongatapu, Ha’apai, Ongo Niuas) using the “*proportion*” command. Differences in demographic characteristics by settings were tested using univariate logistic regression using the “*logistic*” command. Statistical differences in median participant age tested using nonparametric *k*-sample test on the equality-of-medians. Crude associations between demographic data and Ag-positivity were assessed using univariate logistic regression. Variables with *p* < 0·2 on univariable analyses were included in a multivariable logistic regression; variables were sequentially removed to arrive at the most parsimonious model in which variables with *p* < 0·05 were retained. Geographic information systems software ArcGIS Pro (v10.7.1, Environmental Systems Research Institute, Redlands, CA) was used to manage spatial data and produce maps.

### Role of the funding source

The funders of the study had no role in study design, data collection, data analysis, data interpretation, or writing of the report.

## Results

### Demographic characteristics of study participants

**Fig. 3:**
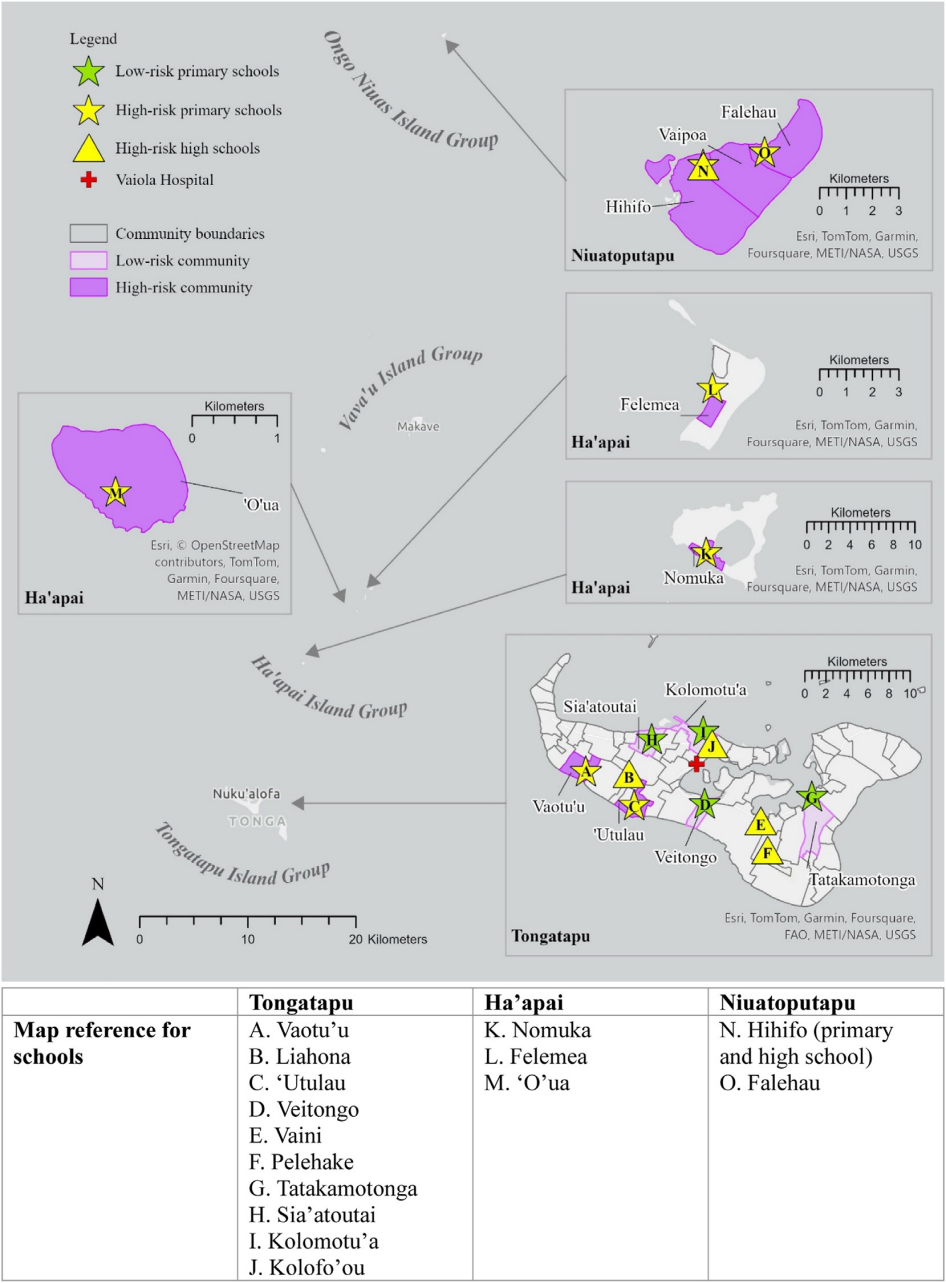
**Map of Vaiola Hospital and selected primary schools, high schools, and communities by island group (Tongatapu, Ha’apai, and Niuatoputapu), Tonga Operational Research for Post-validation Surveillance for Elimination of Lymphatic Filariasis 2024**.

A total of 1787 participants were enrolled from Tongatapu, Ha’apai, and Ongo Niuas, encompassing 12 communities (n = 844), 11 primary schools (n = 416), five high schools (n = 247), diabetes outpatient clinic (n = 220), and volunteers (n = 60) (Table 1 and Fig. 3). The mean participant age was 29·5 years (age range: 5–89 years) and 46% were male. Compared to the community, significantly more males were recruited in schools (primary school odds ratio [OR]: 1·44, *p*-value: 0·002; high school OR: 1·64, *p*-value: 0·001), and fewer males were volunteers (OR: 0·33, *p*-value: 0·001).

**Table 1:** Demographic characteristics of study populations by survey type and study setting, Tonga Operational Research for Post-validation Surveillance for Elimination of Lymphatic Filariasis 2024.

Setting	Island group			Total
	Tongatapu	Ha’apai	Ongo Niuas	
**Community**				
Total communities sampled	6	3	3	12
Total households sampled	104	52	67	223
Total community members sampled	420	190	234	844
Mean households per community (range)	17·6 (14–20)	18·5 (14–22)	22·4 (17–25)	19·2 (14–25)
Mean household size (persons aged ≥ 5 years) (range)	5·7 (1–15)	4·7 (1–9)	4·5 (1–9)	5·2 (1–15)
Mean participants per community (range)	71·9 (45–78)	70·3 (42–92)	78·2 (75–84)	73·3 (42–92)
Mean participant age (range)	33·7 (5–87)	38·3 (5–89)	38·6 (5–79)	36·1 (5–89)
Total male sex (%)	177 (42·1)	86 (45·3)	98 (41·9)	361 (42·8)
**Primary school**				
Total primary schools sampled	6	3	2	11
Total primary students sampled	271	62	83	416
Mean students per school (range)	56·4 (21–86)	25·3 (7–29)	57·2 (16–67)	51·9 (7–86)
Mean student age (range)	9·9 (5–14)	9·9 (6–15)	8·4 (5–12)	9·6 (5–15)
Total male sex (%)	143 (52·8)	32 (51·6)	41 (49·4)	216 (51·9)
**High school**				
Total high schools sampled	4		1	5
Total students sampled	187		60	247
Mean students per school (range)	51·4 (22–60)		60	53·5 (22–60)
Mean student age (range)	17·4 (11–24)		15·7 (13–19)	17·1 (11–24)
Total male sex (%)	112 (59·9)		24 (40·0)	136 (55·1)
**Diabetes clinic**				
Total patients sampled	220			
Mean patient age (range)	56·6 (15–86)			
Total male sex (%)	90 (41·1)			
**Volunteers**				
Total volunteers sampled	49		11	60
Mean volunteers age (range)	26·3 (16–73)		33·4 (23–59)	27·6 (16–73)
Total male sex (%)	8 (16·3)		4 (36·4)	12 (20·0)

### Antigen and microfilariae prevalence

Overall, 92·3% (1787/1955) of the target sample size was enrolled, of whom 1755 (98·2%) were tested using FTS. Thirty-two (1·8%) participants did not have an FTS conducted (because there was insufficient blood collected [n = 24] or difficulty collecting blood [n = 8]). Eighteen FTS were invalid because blood did not flow to the end of the test strip, thus 1737 of 1755 participants had a valid FTS result (Supplementary Fig. S1).

Overall, 39 of 1737 (2·2%; 95% CI: 1·6–3·1) participants were Ag-positive (mean age: 43·5 years; age range: 9–72 years), and 30 of 39 were male. Ag-positive participants were identified from eight of 12 communities, two of 11 primary schools, and one of five high schools. One volunteer tested Ag-positive. No Ag-positives were identified from the clinic-based survey. Seventeen Ag-positive participants were from Ongo Niuas, 17 from Ha’apai, and five from Tongatapu. The highest crude Ag prevalence was in Ha’apai (6·8%; 95% CI: 4·3–10·6), followed by Ongo Niuas (4·4%; 95% CI: 2·8–7·0), and Tongatapu (0·5%; 95% CI: 0·2–1·1).

**Fig. 4:**
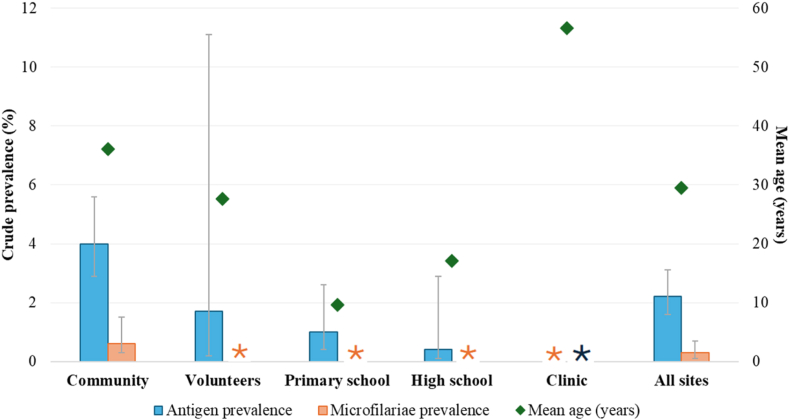
**Antigen and microfilariae crude prevalence estimates and mean participant age stratified by sampling strategy, Tonga Operational Research for Post-validation Surveillance for Elimination of Lymphatic Filariasis 2024.** Asterisks denote zero prevalence. For Mf crude prevalence calculations, Ag-negatives were assumed to be Mf-negative.

By setting, the highest crude Ag prevalence was in communities (4·0%; 95% CI: 2·9–5·6), followed by volunteers (1·7%; 95% CI: 0·2–11·1), primary schools (1·0%; 95% CI: 0·4–2·6), and high schools (0·4%; 95% CI: 0·1–2·9) (Fig. 4). Ten Ag-positive participants had travelled in the past 12 months within Tonga, and one had travelled overseas to Australia/New Zealand.

Slides were made from 34 of 39 Ag-positive blood samples; five of 34 were Mf-positive (mean age: 51·6 years; age range: 30–72 years); all were male and identified in communities. Assuming that Ag-negative participants were Mf-negative, crude Mf prevalence was 0·3% (95% CI: 0·1–0·7) (Fig. 4). One Mf-positive participant had travelled to Australia/New Zealand in the previous 12 months (Table 2).

**Table 2:** Antigen and microfilariae results stratified by survey type, Tonga Operational Research for Post-validation Surveillance for Elimination of Lymphatic Filariasis 2024.

For Mf crude prevalence calculations, Ag-negatives were assumed to be Mf-negative. Cells shaded in light blue indicate ‘low-risk’ communities.

Ag: antigen; FTS: filarial test strip; Mf: microfilariae; NR: No result.

∗Estimated population of ‘O’ua from 2021 census = 56 (less than the target sample size of 75).

ˆVaipoa was added following requests by Tonga MOH and was not classified as a ‘high-risk’ community in the initial site selection. There are only three communities in Niuatoputapu and, given the difficulty in accessing Onga Niuas, Tonga MOH felt it pertinent to test all communities on the island. ∞School teachers, nurses, and community members who asked to be tested were included as volunteers.

^#^One-sided 97·5% binomial exact confidence intervals were calculated for 0% prevalences.

### Community survey

**Fig. 5:**
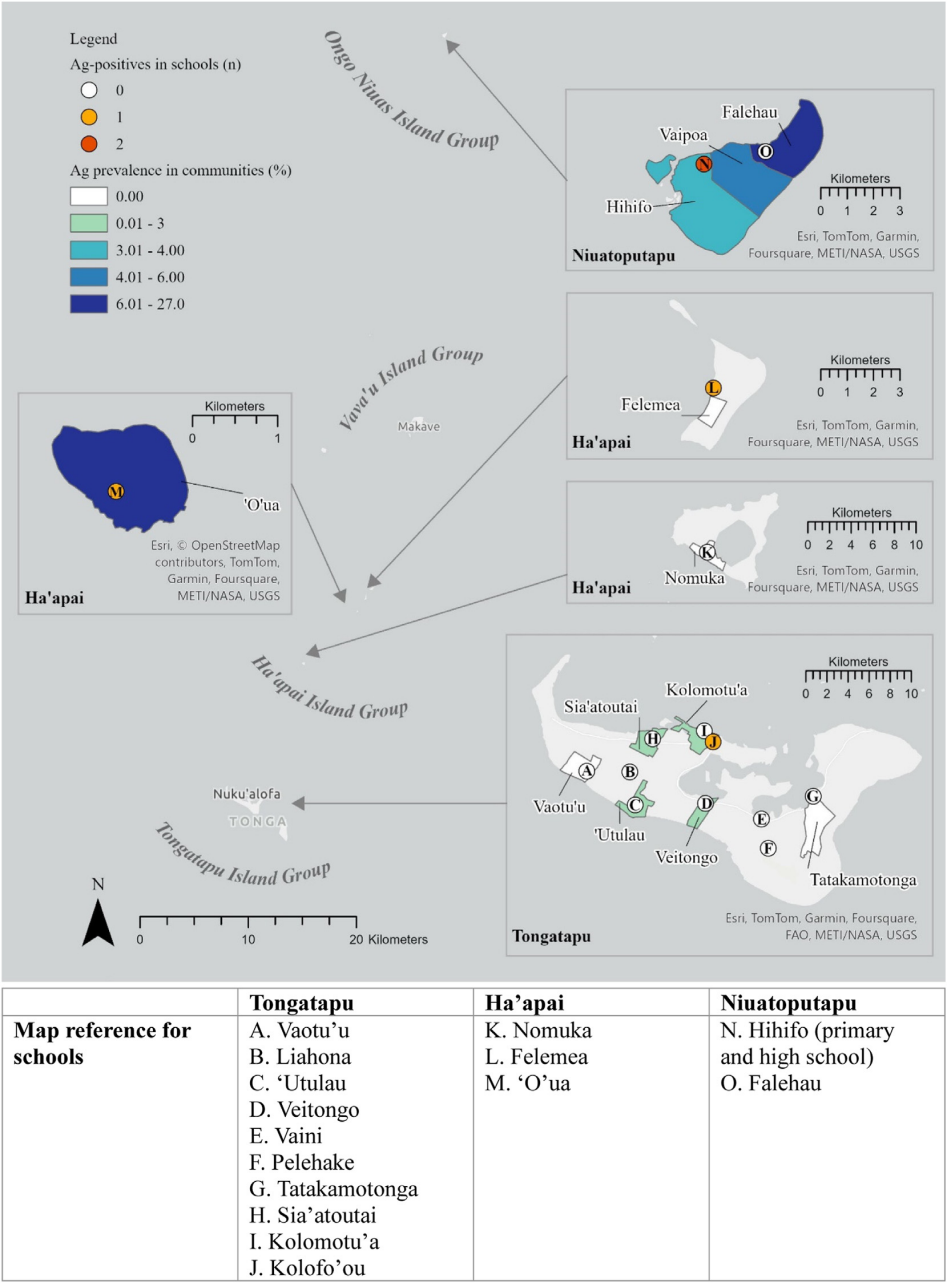
**Crude antigen (Ag) prevalence by community (shading) and total Ag-positive by school (coloured circles) in Tongatapu, Ha’apai, and Niuatoputapu, Tonga Operational Research for Post-validation Surveillance for Elimination of Lymphatic Filariasis 2024**.

From 12 communities, 844 participants (mean age: 36·1 years; age range: 5–89 years; 43·3% male) were recruited, of whom 827 of 844 (98·0%) had a valid FTS result and 33 (4·0%; 95% CI: 2·8–5·6) were Ag-positive. Ag-positives were identified in eight of 12 communities, had a mean age of 48·7 years (age range: 11–72), and 81·8% (95% CI: 64·2–91·9) were male. Fifteen Ag-positives were from Ha’apai, 14 from Ongo Niuas, and four from Tongatapu; crude community-level Ag-prevalence ranged from 1·3% (95% CI: 0·2–8·8) in ‘Utulau to 26·8% (95% CI: 16·8–39·8) in ‘O’ua (Fig. 5).

Mf slides were made from 29 of 33 Ag-positive samples, and five were Mf-positive (mean age: 51·6 years; age range: 30–72 years), all of whom were male. Assuming that Ag-negative participants were also Mf-negative, crude Mf prevalence was 0·6% (95% CI: 0·3–1·5).

### Primary and high school surveys

From 11 schools (six in Tongatapu, three in Ha’apai, and two in Niuatoputapu), 416 primary school students (mean age: 9·6 years, age range: 5–15 years; 51·9% male) were recruited, of whom 404 (97·1%) had a valid FTS result. Four (1·0%; 95% CI: 0·4–2·6) students were Ag-positive (mean age: 9·5 years, age range: 9–11 years; 75·0% male); two were from schools in Ha’apai and two from a school in Niuatoputapu. Crude Ag prevalence ranged from 3·1% (95% CI: 0·8–11·7) in Hihifo to 14·3% (95% CI: 2·0–58·2) in ‘O’ua (Fig. 5). No primary school students were Mf-positive.

In total, 247 students (mean age: 16·8 years, age range: 11–24 years; 55·1% male) were tested at five high schools, four of which were boarding schools. In total, 240 (97·2%) had a valid FTS result and one student from Tongatapu (age: 17; female) tested Ag-positive, giving an overall prevalence of 0·4% (95% CI: 0·1–2·9). Insufficient blood was collected to make slides for Mf (Fig. 5).

### Clinic survey

Overall, 207 of 220 (94·1%) (mean age: 56·5 years, age range: 15–86 years; 41·1% male) participants recruited from the diabetes outpatient clinic at Vaiola Hospital had a valid FTS result. No Ag-positives were found.

### Testing of volunteers

Sixty volunteers were tested for LF, including 25 schoolteachers from surveyed schools (14 from Tongatapu and 11 from Ongo Niuas) and 35 participants from a women’s group in Sia’atoutai community. Overall, 59 (98·3%) had a valid FTS (mean age: 27·6 years, age range: 16–73 years; 20·0% male) and one teacher (age: 37; female) from Ongo Niuas tested Ag-positive, giving an Ag prevalence of 1·7% (95% CI: 0·2–11·1). No Mf was detected.

### Comparison of antigen and microfilariae prevalence between low-risk vs high-risk participants

A total of 1567 participants were recruited from sites considered ‘high-risk’ (n = 1040 participants) and ‘low-risk’ (n = 527 participants). Overall, participants from ‘high-risk’ sites had significantly higher median age (25 vs 13 years; *p* < 0·002). When stratified by site, the median age of participants was higher in ‘high-risk’ vs ‘low-risk’ communities (37·0 vs 25·5 years; *p* < 0·001) and schools (15·0 vs 10·0 years; *p* < 0·001). No significant difference in the total number of male participants was seen (Table 3).

**Table 3:** Summary of demographic characteristics and Ag-positivity among participants from ‘low-risk’ and ‘high-risk’ sites, Tonga Operational Research for Post-validation Surveillance for Elimination of Lymphatic Filariasis 2024.

	Total sites (n)	Total participants (n)	Participant age				Participant sex	
			Median (years)	IQR	Range (years)	*p*-value^a^	Male n (%)	*p*-value^b^
**All sites**								
High-risk	20	1040	18·0	29·0	5–89	0·002	487 (46·8)	0·53
Low-risk	8	527	13·0	20·0	5–87		238 (45·2)	
**Communities**								
High-risk	8	579	37·0	34·0	5–89	<0·001	247 (42·7)	0·51
Low-risk	4	300	25·5	28·5	5–87		121 (40·3)	
**Primary/high schools**								
High-risk	12	461	15·0	9·0	5–59	<0·001	240 (52·1)	0·90
Low-risk	4	227	10·0	3·0	5–54		117 (51·5)	

a Statistical differences in median values tested using nonparametric k-sample test on the equality-of-medians.

b Statistical differences in proportion of males vs females tested using Pearson chi^2^ test.

Eight ‘high-risk’ (n = 579 participants) and four ‘low-risk’ (n = 300 participants) communities were included. Overall, three Ag-positives were identified from three of four ‘low-risk’ communities and 30 Ag-positives were identified from five of eight ‘high-risk’ communities. All five Mf-positives were identified from two of eight high-risk communities. Twelve ‘high-risk’ and four ‘low-risk’ schools were included; no Ag-positives were found in the four ‘low-risk’ schools and six Ag-positives were identified from four of seven ‘high-risk’ primary schools (inclusive of one volunteer teacher) and one of five high schools.

### Associations with Ag-positivity

The prevalence of demographic variables, and their unadjusted and adjusted associations with Ag-positivity are described in Table 4. Multivariable regression found that males vs females (aOR: 4·86; *p* < 0·001), older ages vs 5–10 years (21–50 years aOR: 3·67; *p* = 0·045 and >50 years aOR: 7·51; *p* = 0·002), and residents of Ha’apai (aOR: 15·08; *p* < 0·001) and Ongo Niuas (aOR: 10·85; *p* < 0·001) vs Tongatapu were significantly more likely to be Ag-positive. At univariate analysis, associations between Ag-positivity and ‘high-risk’ communities, primary schools, and high schools were seen, though this association did not persist following adjustment for other variables.

**Table 4:** Summary of demographic factors, and their associations with antigen-positivity on univariable and multivariable logistic regression, Tonga Operational Research for Post-validation Surveillance for Elimination of Lymphatic Filariasis 2024.

	N (%)	OR	*p*-value	aOR	*p*-value
**Age (years)**					
5–10	362 (20·3)	REF	REF	REF	REF
10–20	560 (31·4)	1·07 (0·25–4·51)	0·926	1·36 (0·32–5·82)	0·677
21–50	479 (26·8)	3·58 (1·02–12·57)	0·046	**3·67 (1·03–13·11)**	**0·045**
≥50	384 (21·5)	5·59 (1·62–19·23)	0·006	**7·51 (2·13–26·47)**	**0·002**
**Sex**					
Female	971 (54·4)	REF	REF	REF	REF
Male	815 (45·6)	4·07 (1·92–8·63)	<0·001	**4·86 (2·25–10·46)**	**<0·001**
**Survey type**					
Community	844 (47·3)	REF	REF		
Primary school	416 (23·3)	0·24 (0·08–0·68)	0·008		
High school	247 (13·8)	0·10 (0·01–0·74)	0·024		
Clinic	220 (12·3)	–	–		
Volunteer	60 (3·4)	0·41 (0·06–3·09)	0·390		
**Island group**					
Tongatapu	1147 (64·2)	REF	REF	REF	REF
Ha’apai	252 (14·1)	15·92 (5·82–43·60)	<0·001	**15·08 (5·41–42·05)**	**<0·001**
Ongo Niuas	388 (21·7)	10·13 (3·71–27·64)	<0·001	**10·85 (3·91–30·08)**	**<0·001**
**Risk group**					
Low risk/undefined	747 (41·8)	REF	REF		
High risk	1040 (58·2)	8·64 (2·65–28·18)	<0·001		
**Travel in the past 12 months**					
No/unspecified	1188 (66·5)	REF	REF		
Yes	599 (33·5)	0·99 (0·50–1·94)	0·975		
**Highest level of education**					
None/primary	179 (10·1)	REF	REF		
Secondary or above	939 (52·7)	0·72 (0·31–1·68)	0·451		
Current primary/high school student	663 (37·2)	0·19 (0·05–0·59)	0·004		

Results in bold indicate demographic characteristics significantly associated with Ag-positivity at *p* < 0·05.

## Discussion

Our study identified persistent LF transmission in Tonga with an overall crude Ag and Mf prevalence in our target population of 2·2% and 0·6%, respectively. The last nationwide MDA round in Tonga was in 2005, with targeted MDA in Ongo Niuas in 2006; therefore, we would expect all individuals born after 2006 (now aged ≤ 20 years) to be Ag-negative if transmission had been successfully interrupted. However, eight participants aged ≤ 20 years tested Ag-positive, residing across all three island groups.

Of the four survey types adopted in our study, community surveys yielded the highest Ag prevalence (4·0%) and were the source of all five Mf-positive participants. We targeted ‘high-risk’ communities based on local knowledge and historical survey records and found that participants from these communities had higher odds of being Ag-positive compared to participants from ‘low-risk’ communities. These findings indicate that our methodology was more sensitive than random sampling for identifying persistent LF transmission in Tonga; similar approaches have been used successfully in Thailand’s PVS strategy, where human blood surveys are targeted in areas previously endemic for LF.^13^

Interestingly, the community with the highest Ag prevalence was ‘O’ua (26·8%), which was selected based on high Ag and Mf prevalence recorded in the sentinel, community-based mid-term (B) survey in 2004,^7^ despite no Ag-positive children being identified in this community in any of the school-based TAS’. The effectiveness of school-based TAS for LF surveillance has been questioned, particularly in low transmission settings; studies from Sri Lanka found that both adult-TAS and molecular xenomonitoring were more sensitive than school-based TAS for detecting residual LF following MDA.^14^ These findings corroborate studies in India,^15^ American Samoa,^16^ and Madagascar^17^ that found persistence of LF infection in adults in areas that had met school-based TAS targets. This emphasises the need to review historical and pre-TAS surveys, as well as the most recent school-based TAS, to identify possible LF hotspots for targeted PVS.

Our study is the first to conduct LF surveillance in boarding schools; we considered this approach a convenient PVS strategy that could identify areas of high prevalence if several students from the same island or community were found to be positive. A limitation of this approach is the inability to determine whether Ag-positives were infected at home or at school. We found one Ag-positive boarding school student and four Ag-positive primary school students, and overall, students had significantly lower odds of testing Ag-positive compared to community participants. Despite the low Ag prevalence found in schools, continued school surveillance could be a useful and cost-effective strategy in Tonga, particularly given the large number of boarding school students from outer islands. Research is currently being conducted into the potential use of antifilarial antibody surveillance in school students unexposed to MDA as an indicator of residual LF transmission.^18^ We are not aware of any published school-based PVS activities, but if school-based surveys are a preferred strategy, incorporating antibody testing alongside Ag testing may be a sensitive PVS approach.

We also recorded Ag-positives from ‘low-risk’ communities, though at a significantly lower prevalence than in ‘high-risk’ communities. Finding Ag-positive participants in ‘low-risk’ communities and schools, as well as among volunteers, suggests that a combination of PVS strategies should be considered in Tonga, not only targeted surveys of ‘high-risk’ communities.

Contrastingly, we found zero Ag-positives in our clinic survey. Togo, the first country in sub-Saharan Africa to eliminate LF, established a facility-based surveillance system consisting of i) a laboratory-based surveillance system (from 2006 to 2007) testing blood collected for malaria diagnosis, and ii) testing donated blood from blood banks.^19^ From 2006 to 2011, only three Mf cases were detected, suggesting no ongoing transmission of LF.^19^ However, PVS among migrant groups in Togo found high Ag prevalence, indicating that periodic monitoring of migrants may be a more sensitive surveillance approach than passive laboratory surveillance in this setting.^20^ This raises the question of whether health facility screening is an effective PVS strategy; however, the relative efficiency of PVS strategies will likely vary between countries, and guidance will be required to help countries select the most contextually appropriate PVS approach.

At the time of writing, there was limited guidance available on how best to conduct PVS. WHO’s manual: *Validation of lymphatic filariasis as a public health problem*,^5^ which was released in 2017, i) defines PVS, ii) emphasises that a commitment to PVS must be in place at the time of dossier submission, iii) suggests potential PVS activities (periodic cross-sectional surveys, routine surveillance or target population groups, and molecular xenomonitoring), and iv) details how PVS results should be submitted to WHO.^5^ The manual also encourages programmes to integrate PVS activities with existing health services and coordinate with vector-borne disease programmes for sustainability. However, there are no recommendations on the frequency of PVS activities, the length of time PVS should be conducted for, measurable thresholds to determine whether elimination as a public health problem has been sustained, or how ‘signals’ of persistent transmission should be dealt with (aside from specifying treatment regimens for “communities where post-MDA or PVS identified infection suggesting local transmission”).^21^ It is also not clear whether national commitments to PVS are monitored, or whether countries will be held accountable for not conducting PVS.

For countries such as Tonga, the absence of such detailed guidance has created uncertainties around the need for PVS, or what an appropriate, efficient, and sustainable PVS strategy would be. As a result, PVS had not been conducted in Tonga prior to the current study. We note, however, that PVS guidelines are in development and are anticipated to be published in 2025 in the *WHO 2*nd *Edition of the Lymphatic Filariasis Monitoring & Evaluation manual*.^22^ The manual is likely to recommend that countries use a combination of at least two of the following strategies for PVS: i) health facility screening, ii) integration into existing standardised surveys, iii) targeted surveys of high-risk areas or groups, and iv) molecular xenomonitoring of mosquitoes.^22^

An advantage of our study design was the ability to assess four surveillance strategies, determine their feasibility within the Tongan context, and make evidence-based recommendations to MOH for future LF surveillance. Importantly, two of the strategies assessed (‘health facility screening’ and ‘targeted surveys of high-risk areas or groups’) will likely be recommended by WHO in the upcoming guidelines. As LF is predominantly asymptomatic during the infective stage, PVS should constitute an active surveillance strategy to detect and respond to LF cases in an appropriate and timely manner to prevent resurgence. Our findings suggest that community surveillance of high-risk areas and groups could be an efficient PVS approach for Tonga. Ensuring that males are incorporated in PVS is essential; we found that 82% of Ag-positives and 100% of Mf-positives in our community survey were male. Published literature has also identified associations between Ag-positivity and male sex in many settings, including the Pacific.^11^^,^^16^^,^^23^ We do, however, acknowledge that community surveys have several limitations compared to other surveillance strategies; there is a risk that adults (particularly males) are away from home during the time of survey, preventing an accurate representation of adult males and females. Community surveys can also be logistically more complicated and resource intensive than other surveillance strategies. These challenges are further compounded by limited workforces in many LF-endemic countries, coupled with a plethora of competing public health priorities, which can hinder the prioritisation of community surveys for PVS.

Moving forward, it is important for PVS guidelines to consider contextual limitations, and steps to develop tailored guidelines for Pacific Island Countries and Territories (PICTs) should be considered. Tonga, like many other PICTs, is a very geographically dispersed nation with areas that are hard and expensive to reach, thus the cost and logistical implications for PVS need to be considered. By working with ministries, PVS can be tailored in a culturally appropriate way to include men. This could be done by testing at work sites, after church gatherings, rugby games, community meetings, evening Kava circles etc. In American Samoa, adults of working age were successfully recruited from a work place (a tuna cannery) and tested for LF^23^ and a similar strategy could be adopted in Tonga. Additionally, single-disease, siloed surveillance is not cost-effective; therefore, integrating PVS into the existing health infrastructure is crucial. Adding LF testing to existing standardised surveys (a platform suggested by WHO) could be a means to reduce the costs of conducting an independent survey. We are planning to integrate LF surveillance with an upcoming STEPwise approach to non-communicable disease risk factor surveillance (STEPS) survey in Niue,^24^ and such strategies could be employed in other similar settings. Potentially, including LF as a notifiable disease in PICTs that have eliminated LF could be a way to ensure prevalence continues to be monitored.

A key limitation of our study is that we did not conduct a population representative sample, thus we cannot estimate the overall LF prevalence in Tonga. Furthermore, we only surveyed three of five island groups and LF testing will be required in the remaining island groups to understand the extent of transmission and identify any additional hotspots. Tonga MOH will now need to ascertain whether the Ag prevalence levels justify national or targeted MDA. A treatment plan is currently being discussed between Tonga MOH and WHO, but will likely include individual treatment of Ag-positives found in Tongatapu, and targeted MDA for residents of ‘O’ua and Niuatoputapu.

### Conclusion

Our findings emphasise the need to develop a contextually appropriate and sustainable PVS system in Tonga that can be integrated into the existing health infrastructure. Countries that have eliminated LF as a public health problem need clear guidance about next steps once signals of LF transmission have been identified. As PICTs are leading the way with LF elimination and PVS, they will be the leaders in generating evidence on the effectiveness of surveillance strategies. Future studies should explore culturally appropriate strategies to include older men in integrated PVS strategies, and with a more intense focus in areas of previously high prevalence, to ensure that any LF transmission is identified in a timely manner.

## Contributors

Conceptualisation and funding acquisition: OT, CL, SS. Methodology: OT, JT, RO, CL, SS, HL. Data collection: HL, SW, HJ, BMM, SS. Supervision: OT, CL. Formal analysis: HL. Data interpretation: CL, HL. Writing (original): HL.

HL, HJ, and CL verified the data in the study and take full responsibility for the integrity of the data. All authors had full access to all the data in this study. All authors reviewed and approved the final manuscript.

## Data sharing statement

All relevant data are within the paper. We are unable to provide individual-level antigen prevalence data and demographic data because of the potential for breaching participant confidentiality. The communities in Tonga are very small, and individual-level data such as age, sex, and village of residence could potentially be used to identify specific persons.

## Editor note

The Lancet Group takes a neutral position with respect to territorial claims in published maps and institutional affiliations.

## Declaration of interests

We declare no competing interests.

## Appendix ASupplementary data

Supplementary Table S1

Translated Abstract
