# Supplementary material for: Persistent lymphatic filariasis transmission seven years after validation of elimination as a public health problem: a cross-sectional study in Tonga
Source: Lancet Reg Health West Pac. 2025 Mar 20;57:101513. doi: 10.1016/j.lanwpc.2025.101513 (PMC11987663; doi:10.1016/j.lanwpc.2025.101513)
Supplement: Supplementary Fig. S1 [file mmc1.docx]

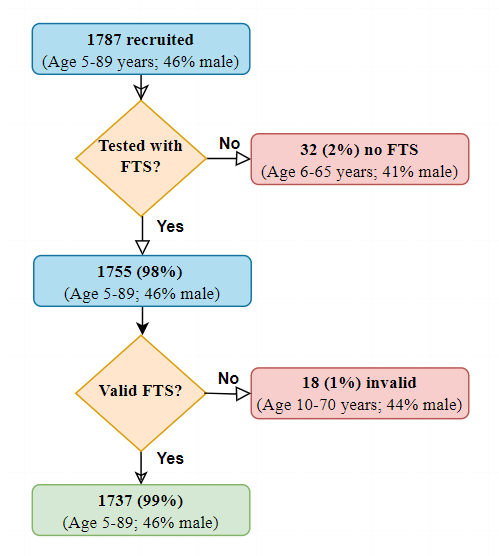


**Supplementary Figure 1: Flow chart of total participants who received a valid Alere™ Filariasis Test Strip (FTS) result, Tonga Operational Research for Post-validation Surveillance for Elimination of Lymphatic Filariasis 2024.**
