# Supplementary material for: Persistent lymphatic filariasis transmission seven years after validation of elimination as a public health problem: a cross-sectional study in Tonga
Source: Lancet Reg Health West Pac. 2025 Mar 20;57:101513. doi: 10.1016/j.lanwpc.2025.101513 (PMC11987663; doi:10.1016/j.lanwpc.2025.101513)
Supplement: Supplementary Table S1 [file mmc2.docx]

**Supplementary Table 1: Study setting and target sample size, Tonga Operational Research for Post-validation Surveillance for Elimination of Lymphatic Filariasis 2024**

| Island group | Setting | Community/school | Reason for inclusion | Target participants* | Total sites | Target sample size |
| --- | --- | --- | --- | --- | --- | --- |
| Tongatapu | ‘High-risk’ communities | Vaotu’u | Households in the communities where Ag-positive school children were identified in TAS  2 in 2011 | Household members aged ≥5 years | 6 | 450 |
|  |  | ‘Utulau |  |  |  |  |
|  | ‘Low-risk’ communities | Veitongo | Communities that recorded no Ag-positive cases in community-based C (Pre-Stop MDA) Survey in 2006 |  |  |  |
|  |  | Tatakamotonga |  |  |  |  |
|  |  | Sia’atoutai |  |  |  |  |
|  |  | Kolomotu’a |  |  |  |  |
|  | ‘High-risk’ primary schools | FWPS ‘Utulau | Ag-positive schools in TAS 2 in 2011 | Grades 5-6 (mostly aged 11-12 years) | 6 | 330 |
|  |  | FWPS Vaotu’u |  |  |  |  |
|  | ‘Low-risk’ primary schools | FWPS Sia’atoutai | No Ag-positive schools identified in TAS 1 (2007), TAS 2 (2011), or TAS 3 (2015), and no Ag-positives recorded in community-based C (Pre-Stop MDA) Survey in 2006 |  |  |  |
|  |  | GPS Kolomotu’a |  |  |  |  |
|  |  | GPS M'ua |  |  |  |  |
|  |  | GPS Veitongo |  |  |  |  |
|  | High schools | Tupou College Tolua | Students, including those boarding from outer islands | Forms 6-7 (mostly aged 16-17) | 4 | 200 |
|  |  | Liahona Middle School |  |  |  |  |
|  |  | Beulah Adventist College |  |  |  |  |
|  |  | Queen Salote College |  |  |  |  |
|  | Diabetes clinic at Vaiola Hospital | Nuku’alofa | Recruitment of chronically ill patients who may have declined medications in previous MDA rounds | Patients presenting to the clinic | 1 | 200 |
| Ha’apai | ‘High-risk’ communities | Nomuka | Households in the communities where Ag-positive school children were identified in TAS  2 in 2011 | Household members aged ≥5 years | 3 | 225 |
|  |  | Felemea |  |  |  |  |
|  |  | ‘O’ua | Community with high Ag prevalence in B (Mid-Term) Survey in 2004 |  |  |  |
|  | ‘High-risk’ primary schools | GPS Mata'aho | Ag-positive schools in TAS 2 in 2011 | Grades 5-6 (mostly aged 11-12 years) | 3 | 165 |
|  |  | FWPS Nomuka |  |  |  |  |
|  |  | GPS ‘O'ua | Located in community with high Ag prevalence in B (Mid-Term) Survey in 2004 |  |  |  |
| Ongo Niuas | ‘High-risk’ communities | Falehau | Households in the communities where Ag-positive school children were identified in TAS  2 in 2011 | Household members aged ≥5 years | 3 | 225 |
|  |  | Hihifo |  |  |  |  |
|  |  | Vaipoa^ | Located on island group with historically high Ag prevalence |  |  |  |
|  | ‘High-risk’ primary schools | FWPS Falehau | Ag-positive schools in TAS 2 in 2011 | Grades 5-6 (mostly aged 11-12 years) | 2 | 110 |
|  |  | GPS Hihifo |  |  |  |  |
|  | ‘High-risk’ high school | Niuatoputapu High School | School located in area of historically high Ag prevalence | Forms 6-7 (mostly aged 16-17) | 1 | 50 |
| TOTAL | | | | | **29** | **1955** |

**In primary schools with low attendance numbers, Grades 3-4 and Forms 1 and 2 were also included. In boarding/high schools with low attendance numbers, Forms 4-5 and Technical and Vocational Education students were also included. ^Vaipoa, Ongo Niuas, was added following requests by Tonga MOH.*

*Ag: Antigen; GPS: Government Primary School; FWPS: Free Wesleyan Primary School; MDA: Mass Drug Administration; TAS: Transmission Assessment Survey.*
