# Supplementary material for: Persistent lymphatic filariasis transmission seven years after validation of elimination as a public health problem: a cross-sectional study in Tonga
Source: Lancet Reg Health West Pac. 2025 Mar 20;57:101513. doi: 10.1016/j.lanwpc.2025.101513 (PMC11987663; doi:10.1016/j.lanwpc.2025.101513)
Supplement: Translated Abstract [file mmc3.docx]

**Kei mafola pe 'a e kulokula fua hili 'a e ta'u 'e fitu mei hono fakangata ko e palopalema ki he mo'ui: ko e fakatotolo na'e fakahoko 'i Tonga**

**FAKAIKIIKI E FAKATOTOLO**

**Puipuitu’a**

Na’e fakapapau’i ‘e he Kautaha Mo’ui ‘a Mamani ko e fonua ‘e 21 kuo fakangata ‘a e hoko e kulokula fua (KF) ko e palopalema ki he mo’ui. ‘Oku kei fiema’u pe ke muimui’i e tu’unga e KF ‘i he ngaahi fonua ko’eni hili hono fakangata; Na’e fakangata e KF ‘i Tonga ‘i he 2017, ka na’e te’eki pe ke fakahoko ha savea talu mei ai. Na’a mau fiema’u ke ‘ilo pe ‘oku kei mafola e KF ‘i Tonga hili hono fakangata ko ha palopalema ki he mo’ui ‘a e kakai.

**Founga**

Na’e fakahoko e savea ‘o fakataumu’a ki he ngaahi feitu’u ‘e 4 ‘i Tongatapu, Ha’apai, mo e Ongo Niua ‘i Me ki Siulai 2024, ‘a ia ko e ngaahi kolo, ‘apiako lautohi si’i, ‘apiako ma’olunga mo e kiliniki fakafaito’o. Ko kinautolu na’e kau ki he savea, na’e tesi kinautolu ki he siemu KF *antigen* (Ag) mo e *microfilariae* (Mf). Ko e ola ‘oku fiema’u mei he savea ke fakamo’oni’i ‘oku kei mafola e KF ko ha sivi Ag ‘oku positivi.

**Ola**

‘I he ‘aho 9 Me ki he 19 Siulai 2024, na’e kau e kakai ‘e toko 1787 ki he savea mei he ngaahi tukui kolo ‘e 12, lautohi si’i ‘e 11, ako ma’olunga ‘e 5, pea mo e kiliniki ‘e 1. ‘I hono fakakatoa, ko e toko 39 (2.2%) na’e Ag-positivi, pea 5 (0.3%) na’e Mf-positivi. Ko e tokolahi taha ‘o e kau Ag-positivi mei he ngaahi kolo (4.0%; 95%CI:2.9-5.6). Ko e kotoa e kau Mf-positivi mei he ngaahi kolo. Ko e kau Ag-positivi na’e ‘i ai ‘enau felave’i mo e kakai tangata (adjusted odds ratio [aOR]:4·86; *p*<0·001), ta’u matu’otu’a ange (> ta’u 50 vs ta’u 5-10 [aOR:7·51; *p*=0·002]), pea nau nofo ‘i Ha’apai (aOR:15·08; *p*<0·001) mo e Ongo Niuas (aOR:10·85; *p*<0·001).

**Faka’uhinga**

Na’e mahino ‘oku kei mafola pe ‘a e KF ‘i Tonga. Ko e savea he ngaahi kolo na’e tokolahi taha ai e kau positivi Ag mo e Mf. ‘Oku fiema’u ke fokotu’u ‘e he Potungaue Mo’ui ha fa’unga ke hokohoko mo tu’uloa hono fai e savea KF ‘o fakatefito he ngaahi koló ke ma’u ai e kakai tangata matu’otu’a ange.

**Fakapa’anga**

Na’e fakapa’anga e savea ‘e he Task Force for Global Health, Bill & Melinda Gates Foundation, and the United States Agency for International Development.
